# Intraoperative Local Administration of Platelet-Rich Plasma (PRP) during Neurolysis Surgery for the Treatment of Digital Nerve Crush Injury

**DOI:** 10.1155/2018/1275713

**Published:** 2018-09-20

**Authors:** Akira Ikumi, Yuki Hara, Eriko Okano, Sho Kohyama, Norihito Arai, Yu Taniguchi, Hisashi Sugaya, Tomokazu Yoshioka, Akihiro Kanamori, Masashi Yamazaki

**Affiliations:** Department of Orthopaedic Surgery, Faculty of Medicine, University of Tsukuba, 1-1-1 Tennoudai, Tsukuba, Ibaraki 3058575, Japan

## Abstract

The digital nerves are important for normal hand function. In addition to conventional therapies such as neurolysis, direct repair, and auto/allografts, new treatments administering growth factors and cells for promoting nerve regeneration exist. Platelet-rich plasma (PRP), an autologous product with proven therapeutic effects for musculoskeletal disorders, is a new treatment option for peripheral nerve injury. We hypothesized that PRP could stimulate healing of digital nerve injuries. In the current case report, intraoperative local administration of PRP was performed during neurolysis surgery for a healthy 28-year-old woman with digital nerve crush injury. Five weeks postinjury, surgery was performed due to severe uncontrollable neuropathic pain and no sensory nerve action potential derivation of the index finger. Therapeutic effects were assessed by physical examination, visual analog scale for pain, and nerve conduction study. Postoperatively, early neuropathic pain relief and good functional recovery were obtained with no PRP-related adverse events. This case report demonstrates the therapeutic potential of intraoperative PRP to enhance the healing process of nerve crush injury in the acute phase and to decrease the neuropathic pain, thus enhancing healing of peripheral nerve crush injury.

## 1. Introduction

The digital nerves are important for normal hand function, given the role of the hand as a distinctive and accurate sensitive organ. Digital nerves are often injured in hand traumas [1]. Once the digital nerve is damaged, sensory disturbance beyond the injured part occurs, which hinders delicate movements of the hand (such as pinching small objects and hanging buttons). Neuropathic pain also occurs after peripheral nerve injury. Neuropathic pain is defined as a type of pain related to the injury and/or dysfunction of the peripheral or central nervous system or alterations in the stimulation of these structures. The pathophysiology of neuropathic pain is not fully understood [2].

The treatment of digital nerve injury depends on the degree of damage. In the case of incomplete neurotmesis such as crush injury, conservative therapy is usually selected because natural recovery can be expected. In the case of complete neurotmesis due to injury from a sharp-edged tool, epineural suturing under the microscope is usually performed. Autologous nerve grafting or artificial nerve transplantation are performed if the case involves nerve defects or in chronic cases whereby direct suturing is difficult after stump refreshment. In the case of incomplete neurotmesis with nerve disorders such as sensory disturbance or neuropathic pain due to adhesion or fibrosis around the nerve, neurolysis is occasionally performed. Although the digital nerve consists only of sensory nerves and motion impairment does not occur following injury, prolongation of neuropathic pain after injury is a problem that needs to be resolved.

In recent years, positive effects of platelet-rich plasma (PRP) on tissue regeneration have been reported [3–10]. PRP is used as an autologous cell-free therapy and contains many bioactive factors of plasma and *α*-granules of platelets, which are involved in wound healing and tissue repair [3, 11–15]. The tissue repair effects of PRP are caused by various growth factors (GFs) in the plasma and *α*-granules of platelets. PRP contains platelet-derived growth factor (PDGF), insulin-like growth factor (IGF), fibroblast growth factor (FGF), transforming growth factor-*β* (TGF-*β*), vascular endothelial growth factor (VEGF), and epidermal growth factor (EGF), which promote cell proliferation and migration [3]. These GFs are also associated with peripheral nerve regeneration. In this regard, the positive effects of PRP on nerve regeneration and evidence of its neuroprotective, neurogenic, and neuroinflammatory effects have been reported in several studies [15–20].

Previous studies have not reported on intraoperative injection for a patient with peripheral nerve injury. Hence, in this current case report, intraoperative local administration of PRP was performed during neurolysis surgery for a patient with digital nerve crush injury. Early neuropathic pain relief and functional recovery were evaluated. This study was conducted with the approval of the Ethics Committee of the Tsukuba University Faculty of Medicine.

## 2. Case Presentation

### 2.1. Case Description

The patient was a healthy 28-year-old woman who works as a book binder with no history of interest to this report. The fingers of her dominant hand were caught in a binding machine during work. Immediately after the injury, sensory disturbance and numbness occurred and persisted. Furthermore, she experienced severe neuropathic pain and anesthesia of her index finger. The patient came to our clinic 2 weeks after the injury.

**Figure 1:**
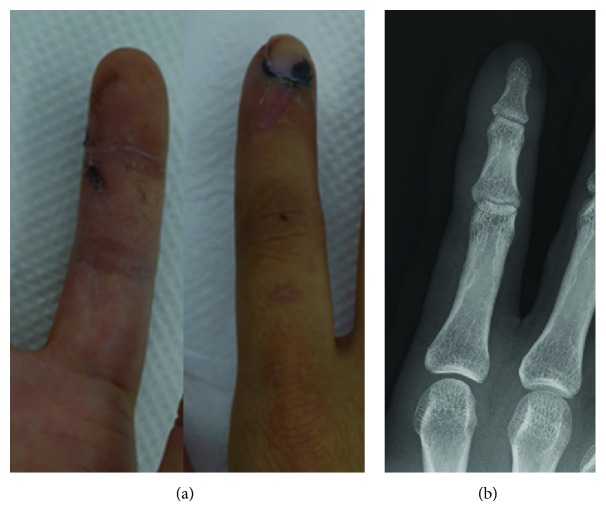
Macro and radiological findings. (a) Macrofinding at 2 weeks after injury. A scar around the ulnar side of the distal interphalangeal (DIP) joint and submaxillary hematoma in index finger was observed. (b) X-ray finding. No fracture was observed.

**Figure 2:**
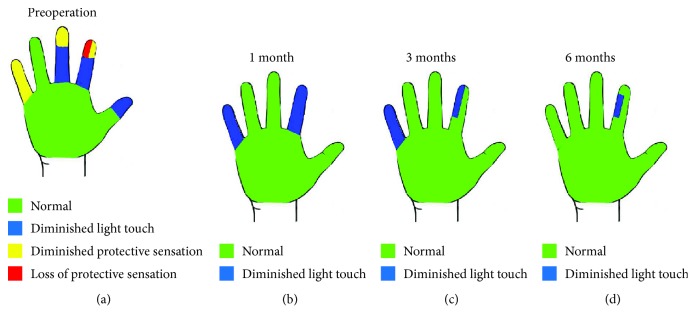
Postoperative course of Semmes-Weinstein monofilament test. (a) Preoperative period. Ulnar side of the apex of the index finger had no sensation. (b) 1 month after surgery. Sensory disturbance recovered. (c) 3 months after surgery. (d) 6 months after surgery. The entire area with the exception of the injured site appeared normal.

A wound scar was observed near the distal interphalangeal (DIP) joint of the ulnar side of the index finger (Figure 1(a)). Sensory examination revealed hypoesthesia in the thumb, index, middle, and little fingers. In the index finger, in particular, the area of the ulnar side beyond the wound was red (indicating loss of protective sensation) based on Semmes-Weinstein monofilament score used by tactile and contact force tester (2SA01, Kono Seisakusho Co. Ltd., Japan) [21] (Figure 2(a)). Tinel's sign was observed, consistent with the wound. Severe neuropathic pain was observed both at rest and during movement. The visual analog scale (VAS) for pain was 10/10 mm. There were no radiological findings suggestive of fracture (Figure 1(b)). The range of motion of the index finger (injured/healthy side) was restricted due to neuropathic pain: metacarpophalangeal (MP) joint: 0°/70°, proximal interphalangeal (PIP) joint: 0°/40°, and DIP joint: 0°/60°. The total active range of motion (TAM) was 63%.

**Figure 3:**
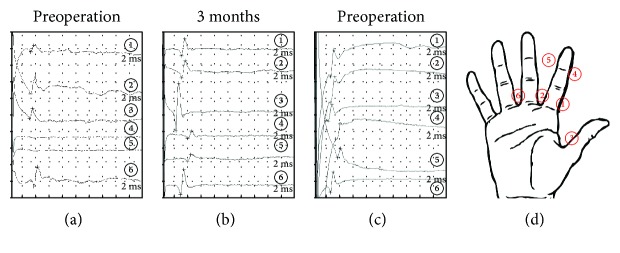
Results of the sensory nerve conduction study. Sensory nerve action potentials (SNAPs) were collected using the antegrade recording method by applying a stimulation to the digital nerve and deriving SNAPs from the forearm just above the median nerve with a surface electrode. (a) Preoperative period. No SNAPs were observed following stimulation at both sides of the index finger beyond the scar. (b) 3 months after surgery. SNAP was observed in each area. (c) 6 months after surgery. Sensory nerve conduction velocity increased compared with that 3 months after surgery. (d) The stimulation point.

In a sensory nerve conduction study (NCS), we attempted to derive sensory nerve action potentials (SNAPs) of the thumb, index, and middle fingers. Using the antegrade recording method, SNAPs were collected by stimulating the digital nerve; they were derived from the forearm just above the median nerve using a surface electrode. No derivation of SNAP was observed in the distal area of the index finger (Figure 3(a)). We also attempted to use ultrasound for diagnosis, but definitive diagnosis proved difficult due to the small size of the digital nerve.

**Figure 4:**
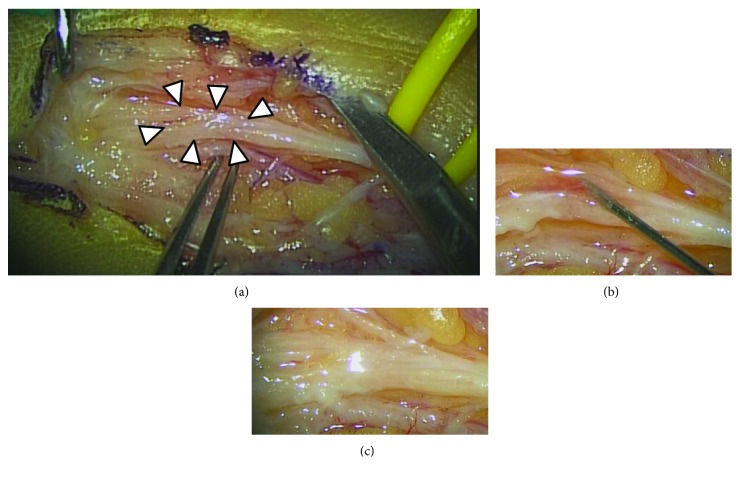
Intraoperative findings. (a) After neurolysis, severe adhesion was observed at the scar area (arrowhead). (b) Intrafascicular injection of platelet-rich plasma (PRP) using a 25-gauge needle. (c) After PRP injection.

Neurotmesis was suspected based on clinical findings and NCS. Although we attempted to control her neuropathic pain, her symptoms were uncontrollable by conservative therapy. If neurotmesis was not denied by NCS, we recommended the patient to conduct intraoperative diagnosis in our facility. Surgery under general anesthesia was performed 5 weeks after injury. Intraoperative findings revealed severe adhesion around the digital nerve, but the nerve preserved its continuity. The intraoperative diagnosis was nerve crush injury. After undergoing neurolysis, 0.5 ml of PRP was intraneurally injected at two locations of the injured area that was identified based on intraoperative findings (total administration volume = 1 ml) (Figure 4). A 25-gauge needle was used for infiltration, and PRP was injected from the proximal side of the injured site under direct vision.

**Figure 5:**
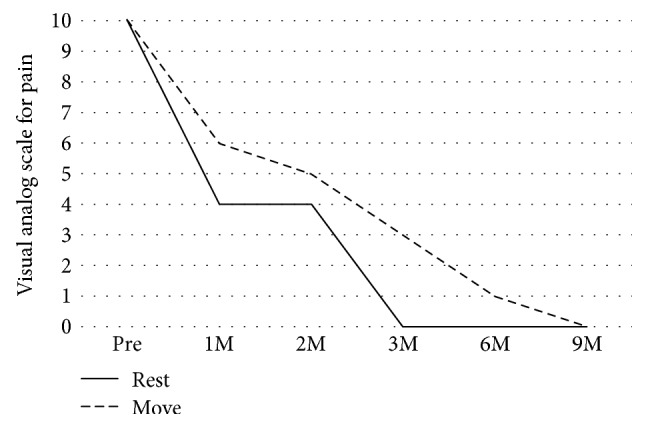
Postoperative course of visual analog scale (VAS) for pain.

After 1 week of immobilization, rehabilitation of active finger motion was initiated. A decrease in neuropathic pain was observed immediately after surgery (Figure 5). Improvement of restricted finger range of motion was recognized from 2 weeks postoperatively and became normal 4 weeks after surgery. In the Semmes-Weinstein monofilament test, the sensory disorder recovered gradually and completely recovered 6 months postoperatively (Figure 2(b)–(d)). The derivation of SNAP was confirmed 3 months postoperatively, and it became normal 9 months postoperatively (Figures 3(b) and 3(c)). Neuropathic pain disappeared 9 months after surgery.

### 2.2. PRP Preparation

PRP was elaborated according to PRGF-Endoret technology (BTI Biotechnology Institute, Vitoria-Gasteiz, Spain) in the cell-processing factory unit of our hospital which fulfills the criteria for good manufacturing product (GMP). Briefly, a total of 36 ml of peripheral venous blood was withdrawn into four tubes of 9 ml containing 3.8% (*w*/*v*) sodium citrate. Blood was centrifuged at 580 *g* for 8 minutes at room temperature (24–26°C). The upper volume of plasma (platelet-poor plasma; PPP), which contains a similar platelet count to that of peripheral blood, was drawn off and discarded in a collection tube. The 2 ml plasma fraction (PRP) located immediately above the sediment of red blood cells, but not including the buffy coat, was collected from each tube (total 8 ml PRP) and transferred to another tube. This tube was taken to the operation room ready for use. This plasma contains a moderate enrichment of platelets (two- to threefold the platelet count of peripheral blood) with scarce leukocytes, being a P2-x-B*β* PRP according to the classification system proposed by DeLong et al. [22]. The concentration rate of administered PRP in this case was 2.23 (Table 1). To initiate clotting, calcium chloride (10% *w*/*v*) was added to the liquid PRP aliquots just before injection. All procedures were performed under sterile conditions.

## 3. Discussion

Treatment for peripheral nerve injury is one of the difficult tasks in the field of orthopedic and plastic surgery. Although microsurgical technology and the development of artificial nerves are improving, the outcomes are still based on patient age, cause and level of the lesion, complications, duration of denervation, length of nerve defect, nerve repair method, and the experience of the surgeon. Thus, there are cases with unsatisfactory outcomes after surgery [23]. Especially for digital nerve injury, poor outcomes have been obtained after repair surgery; 40% of patients complained of persistent hyperesthesia for up to 2 years. Furthermore, normal sensation was lost [24], and duration for recovery of sensation and improvement in sensitivity were very prolonged [25].

To improve the outcomes of nerve injury after surgical treatment, it is necessary to develop new technologies to promote nerve regeneration after nerve repair surgery. In recent years, attempts have been made to stimulate nerve growth by adding neurotrophic factors or specific cells such as Schwann cells and/or stem cells into the nerve conduit or fibrin glue or to stimulate the innervated muscle by intramuscular injection of growth factors [26, 27]. As a candidate to promote nerve regeneration, we focused on PRP. PRP is defined as the plasma layer fraction containing a large amount of platelets and various growth factors present in plasma and *α*-granules of platelets obtained by centrifuging whole blood. These growth factors act in a complex manner to modulate tissue regeneration upon local administration of PRP to the injured tissue. In recent years, PRP has been used for various tissue injuries in the field of regenerative medicine because of its convenience and safety. Regarding peripheral nerve regeneration, evidence of its neuroprotective, neurogenic, and neuroinflammatory effects have accumulated through basic research and clinical trials [26, 28, 29]. Moreover, recovery of sensory and motor functional neuromuscular units has been reported following PRP treatment [19, 29–32].

PRP is usually classified into leukocyte-rich PRP, which contains leukocytes, and pure PRP, which does not contain leukocytes, depending on preparation conditions [33]. The mechanism of action on tissue regeneration differs depending on the presence or absence of leukocytes. Although the variation and concentration of contained growth factors in PRP influence the degree of tissue regeneration, studies involving suitable preparations for peripheral nerve regeneration have not been conducted in the past. In this case, PRP was prepared using PRGF system IV (BTI Institute, Spain) which has been observed to have a facilitatory effect on nerve regeneration by basic research and clinical case reports [34].

PRP was locally administered into the intrafascicular and epineural spaces during surgery in this case. Immediately after surgery, the restriction of finger range of motion due to neuropathic pain was reduced and normalized 1 month after surgery. Neuropathic pain has been reported to be improved by local administration of PRP, although the mechanism has yet to be elucidated [20, 30, 35]. PRP may reduce postoperative neuropathic pain, an effect that may work effectively when performing postoperative rehabilitation. In recent years, ultrasound-guided injections for intrafascicular and epineural spaces of nerves have been reported [20]. Furthermore, the ultrasound-guided procedure of percutaneous hydrodissection of the median nerve away from the deep surface of the flexor retinaculum can separate a potential soft tissue adhesion from the nerve [36]. Thus, if nerve crush injury was definitively diagnosed before surgery in this case, less invasive treatment using ultrasound-guided techniques could have been performed.

No study has reported the amount of PRP required to promote nerve regeneration. PRP was administered into the intrafascicular space of the digital nerve in this case. Intraoperative findings suggested that administration of over 1 ml PRP infiltration results in pressure on the nerve fascicles in the perineurium because 1 ml of PRP infiltration causes the digital nerve width to increase to twice as large as that before injection. Furthermore, leakage of PRP from the injection point was already observed when we administrated 1 ml of the solution. Therefore, we did not administer additional PRP. Zheng et al. reported that the difference in the platelet concentration of PRP induced differing effects on the proliferation of Schwann cells in vitro [32]. They showed that the proliferative effects of PRP for Schwann cells decreased at high concentrations. Their results suggested that platelet concentration and the amount of administrated PRP affects the therapeutic effects on the regeneration of each tissue. Thus, it is important to verify the appropriate platelet concentration rate and amount of PRP necessary for nerve regeneration.

In conclusion, intraoperative local administration of PRP to an injured nerve promotes functional recovery with no adverse events due to PRP. PRP may therefore be safely used in patients with peripheral nerve injury.

## Figures and Tables

**Table 1:** The amount of blood components.

	White blood cell count (/*μ*l)	Hemoglobin (g/dl)	Platelet (×10^4^/*μ*l)
Whole blood	7000	13.7	27.1
Platelet-poor plasma	0	0.0	32.5
Platelet-rich plasma	0	0.1	60.4
